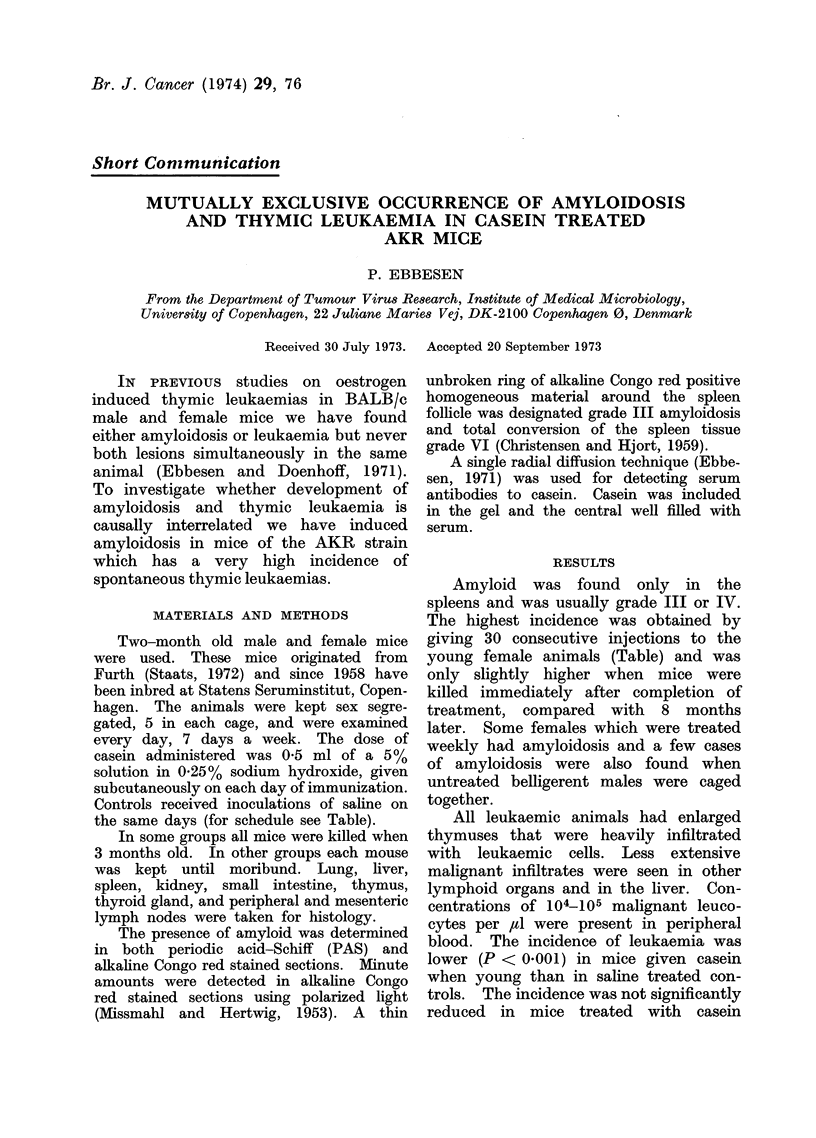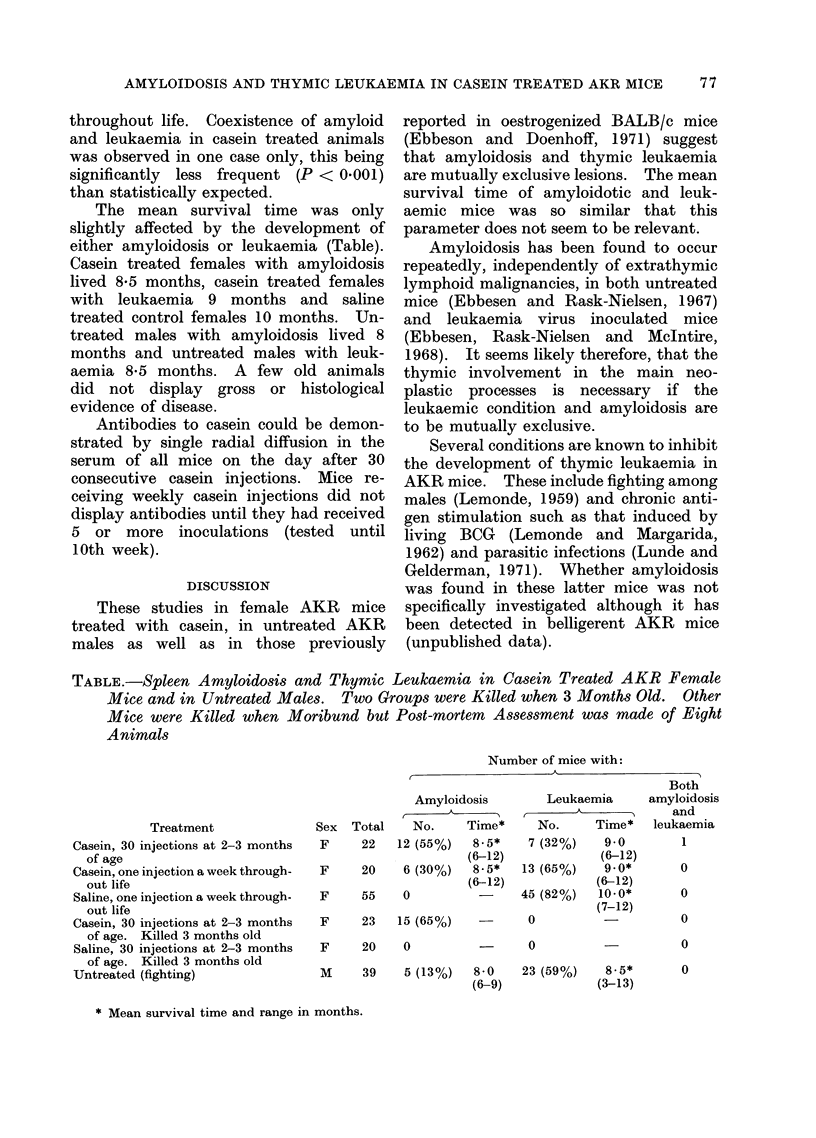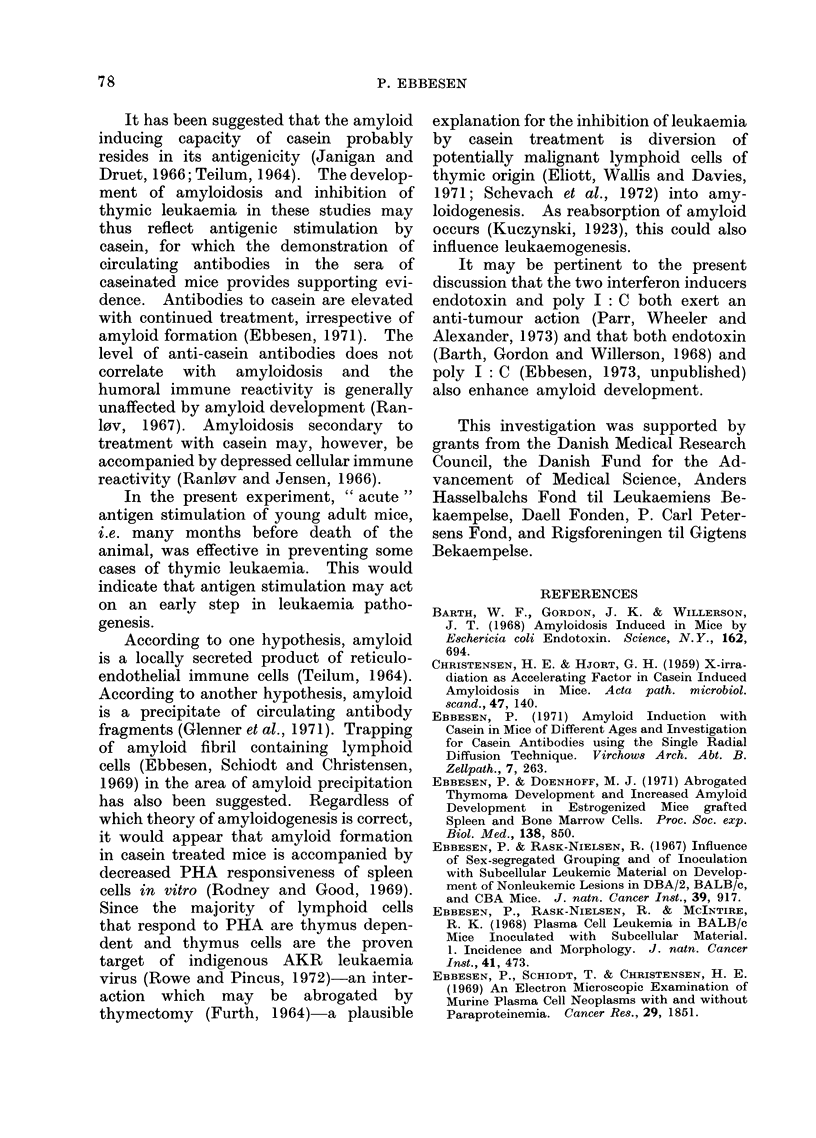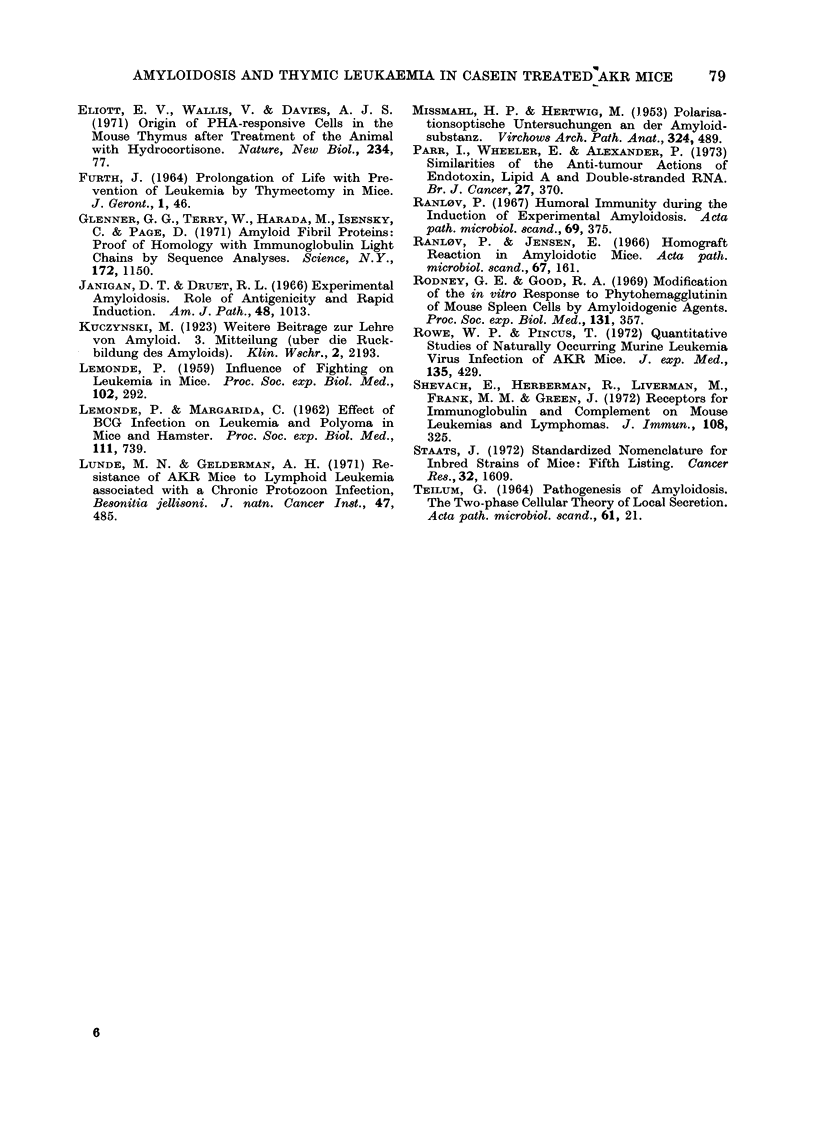# Mutually exclusive occurrence of amyloidosis and thymic leukaemia in casein treated AKR mice.

**DOI:** 10.1038/bjc.1974.10

**Published:** 1974-01

**Authors:** P. Ebbesen


Br. J. Cancer (1974) 29, 76

### *Short Communication*

### MUTUALLY EXCLUSIVE OCCURRENCE OF AMYLOIDOSIS AND THYMIC LEUKAEMIA IN CASEIN TREATED AKR MICE

P. EBBESEN

*From the Department of Tumour Virus Research, Institute of Medical Microbiology, University of Copenhagen, 22 Juliane Maries Vej, DK-2100 Copenhagen Ø, Denmark*

Received 30 July 1973. Accepted 20 September 1973

IN PREVIOUS studies on oestrogen induced thymic leukaemias in BALB/c male and female mice we have found either amyloidosis or leukaemia but never both lesions simultaneously in the same animal (Ebbesen and Doenhoff, 1971). To investigate whether development of amyloidosis and thymic leukaemia is causally interrelated we have induced amyloidosis in mice of the AKR strain which has a very high incidence of spontaneous thymic leukaemias.

### MATERIALS AND METHODS

Two-month old male and female mice were used. These mice originated from Furth (Staats, 1972) and since 1958 have been inbred at Statens Seruminstitut, Copenhagen. The animals were kept sex segregated, 5 in each cage, and were examined every day, 7 days a week. The dose of casein administered was 0·5 ml of a 5% solution in 0·25% sodium hydroxide, given subcutaneously on each day of immunization. Controls received inoculations of saline on the same days (for schedule see Table).

In some groups all mice were killed when 3 months old. In other groups each mouse was kept until moribund. Lung, liver, spleen, kidney, small intestine, thymus, thyroid gland, and peripheral and mesenteric lymph nodes were taken for histology.

The presence of amyloid was determined in both periodic acid–Schiff (PAS) and alkaline Congo red stained sections. Minute amounts were detected in alkaline Congo red stained sections using polarized light (Missmahl and Hertwig, 1953). A thin unbroken ring of alkaline Congo red positive homogeneous material around the spleen follicle was designated grade III amyloidosis and total conversion of the spleen tissue grade VI (Christensen and Hjort, 1959).

A single radial diffusion technique (Ebbesen, 1971) was used for detecting serum antibodies to casein. Casein was included in the gel and the central well filled with serum.

### RESULTS

Amyloid was found only in the spleens and was usually grade III or IV. The highest incidence was obtained by giving 30 consecutive injections to the young female animals (Table) and was only slightly higher when mice were killed immediately after completion of treatment, compared with 8 months later. Some females which were treated weekly had amyloidosis and a few cases of amyloidosis were also found when untreated belligerent males were caged together.

All leukaemic animals had enlarged thymuses that were heavily infiltrated with leukaemic cells. Less extensive malignant infiltrates were seen in other lymphoid organs and in the liver. Concentrations of $10^4$–$10^5$ malignant leucocytes per $\mu$l were present in peripheral blood. The incidence of leukaemia was lower ($P < 0{\cdot}001$) in mice given casein when young than in saline treated controls. The incidence was not significantly reduced in mice treated with casein throughout life. Coexistence of amyloid and leukaemia in casein treated animals was observed in one case only, this being significantly less frequent ($P < 0{\cdot}001$) than statistically expected.

The mean survival time was only slightly affected by the development of either amyloidosis or leukaemia (Table). Casein treated females with amyloidosis lived 8·5 months, casein treated females with leukaemia 9 months and saline treated control females 10 months. Untreated males with amyloidosis lived 8 months and untreated males with leukaemia 8·5 months. A few old animals did not display gross or histological evidence of disease.

Antibodies to casein could be demonstrated by single radial diffusion in the serum of all mice on the day after 30 consecutive casein injections. Mice receiving weekly casein injections did not display antibodies until they had received 5 or more inoculations (tested until 10th week).

### DISCUSSION

These studies in female AKR mice treated with casein, in untreated AKR males as well as in those previously reported in oestrogenized BALB/c mice (Ebbeson and Doenhoff, 1971) suggest that amyloidosis and thymic leukaemia are mutually exclusive lesions. The mean survival time of amyloidotic and leukaemic mice was so similar that this parameter does not seem to be relevant.

Amyloidosis has been found to occur repeatedly, independently of extrathymic lymphoid malignancies, in both untreated mice (Ebbesen and Rask-Nielsen, 1967) and leukaemia virus inoculated mice (Ebbesen, Rask-Nielsen and McIntire, 1968). It seems likely therefore, that the thymic involvement in the main neoplastic processes is necessary if the leukaemic condition and amyloidosis are to be mutually exclusive.

Several conditions are known to inhibit the development of thymic leukaemia in AKR mice. These include fighting among males (Lemonde, 1959) and chronic antigen stimulation such as that induced by living BCG (Lemonde and Margarida, 1962) and parasitic infections (Lunde and Gelderman, 1971). Whether amyloidosis was found in these latter mice was not specifically investigated although it has been detected in belligerent AKR mice (unpublished data).

TABLE.—*Spleen Amyloidosis and Thymic Leukaemia in Casein Treated AKR Female Mice and in Untreated Males. Two Groups were Killed when 3 Months Old. Other Mice were Killed when Moribund but Post-mortem Assessment was made of Eight Animals*

| | | | Number of mice with: | | | | |
|---|---|---|---|---|---|---|---|
| | | | Amyloidosis | | Leukaemia | | Both amyloidosis and leukaemia |
| Treatment | Sex | Total | No. | Time* | No. | Time* | |
| Casein, 30 injections at 2–3 months of age | F | 22 | 12 (55%) | 8·5* (6–12) | 7 (32%) | 9·0 (6–12) | 1 |
| Casein, one injection a week throughout life | F | 20 | 6 (30%) | 8·5* (6–12) | 13 (65%) | 9·0* (6–12) | 0 |
| Saline, one injection a week throughout life | F | 55 | 0 | — | 45 (82%) | 10·0* (7–12) | 0 |
| Casein, 30 injections at 2–3 months of age. Killed 3 months old | F | 23 | 15 (65%) | — | 0 | — | 0 |
| Saline, 30 injections at 2–3 months of age. Killed 3 months old | F | 20 | 0 | — | 0 | — | 0 |
| Untreated (fighting) | M | 39 | 5 (13%) | 8·0 (6–9) | 23 (59%) | 8·5* (3–13) | 0 |

* Mean survival time and range in months.

It has been suggested that the amyloid inducing capacity of casein probably resides in its antigenicity (Janigan and Druet, 1966; Teilum, 1964). The development of amyloidosis and inhibition of thymic leukaemia in these studies may thus reflect antigenic stimulation by casein, for which the demonstration of circulating antibodies in the sera of caseinated mice provides supporting evidence. Antibodies to casein are elevated with continued treatment, irrespective of amyloid formation (Ebbesen, 1971). The level of anti-casein antibodies does not correlate with amyloidosis and the humoral immune reactivity is generally unaffected by amyloid development (Ranløv, 1967). Amyloidosis secondary to treatment with casein may, however, be accompanied by depressed cellular immune reactivity (Ranløv and Jensen, 1966).

In the present experiment, " acute " antigen stimulation of young adult mice, *i.e.* many months before death of the animal, was effective in preventing some cases of thymic leukaemia. This would indicate that antigen stimulation may act on an early step in leukaemia pathogenesis.

According to one hypothesis, amyloid is a locally secreted product of reticuloendothelial immune cells (Teilum, 1964). According to another hypothesis, amyloid is a precipitate of circulating antibody fragments (Glenner *et al.*, 1971). Trapping of amyloid fibril containing lymphoid cells (Ebbesen, Schiodt and Christensen, 1969) in the area of amyloid precipitation has also been suggested. Regardless of which theory of amyloidogenesis is correct, it would appear that amyloid formation in casein treated mice is accompanied by decreased PHA responsiveness of spleen cells *in vitro* (Rodney and Good, 1969). Since the majority of lymphoid cells that respond to PHA are thymus dependent and thymus cells are the proven target of indigenous AKR leukaemia virus (Rowe and Pincus, 1972)—an interaction which may be abrogated by thymectomy (Furth, 1964)—a plausible explanation for the inhibition of leukaemia by casein treatment is diversion of potentially malignant lymphoid cells of thymic origin (Eliott, Wallis and Davies, 1971; Schevach *et al.*, 1972) into amyloidogenesis. As reabsorption of amyloid occurs (Kuczynski, 1923), this could also influence leukaemogenesis.

It may be pertinent to the present discussion that the two interferon inducers endotoxin and poly I : C both exert an anti-tumour action (Parr, Wheeler and Alexander, 1973) and that both endotoxin (Barth, Gordon and Willerson, 1968) and poly I : C (Ebbesen, 1973, unpublished) also enhance amyloid development.

This investigation was supported by grants from the Danish Medical Research Council, the Danish Fund for the Advancement of Medical Science, Anders Hasselbalchs Fond til Leukaemiens Bekaempelse, Daell Fonden, P. Carl Petersens Fond, and Rigsforeningen til Gigtens Bekaempelse.

### REFERENCES

Barth, W. F., Gordon, J. K. & Willerson, J. T. (1968) Amyloidosis Induced in Mice by *Eschericia coli* Endotoxin. *Science, N.Y.*, **162**, 694.

Christensen, H. E. & Hjort, G. H. (1959) X-irradiation as Accelerating Factor in Casein Induced Amyloidosis in Mice. *Acta path. microbiol. scand.*, **47**, 140.

Ebbesen, P. (1971) Amyloid Induction with Casein in Mice of Different Ages and Investigation for Casein Antibodies using the Single Radial Diffusion Technique. *Virchows Arch. Abt. B. Zellpath.*, **7**, 263.

Ebbesen, P. & Doenhoff, M. J. (1971) Abrogated Thymoma Development and Increased Amyloid Development in Estrogenized Mice grafted Spleen and Bone Marrow Cells. *Proc. Soc. exp. Biol. Med.*, **138**, 850.

Ebbesen, P. & Rask-Nielsen, R. (1967) Influence of Sex-segregated Grouping and of Inoculation with Subcellular Leukemic Material on Development of Nonleukemic Lesions in DBA/2, BALB/c, and CBA Mice. *J. natn. Cancer Inst.*, **39**, 917.

Ebbesen, P., Rask-Nielsen, R. & McIntire, R. K. (1968) Plasma Cell Leukemia in BALB/c Mice Inoculated with Subcellular Material. 1. Incidence and Morphology. *J. natn. Cancer Inst.*, **41**, 473.

Ebbesen, P., Schiodt, T. & Christensen, H. E. (1969) An Electron Microscopic Examination of Murine Plasma Cell Neoplasms with and without Paraproteinemia. *Cancer Res.*, **29**, 1851.

ELIOTT, E. V., WALLIS, V. & DAVIES, A. J. S. (1971) Origin of PHA-responsive Cells in the Mouse Thymus after Treatment of the Animal with Hydrocortisone. *Nature, New Biol.*, **234**, 77.

FURTH, J. (1964) Prolongation of Life with Prevention of Leukemia by Thymectomy in Mice. *J. Geront.*, **1**, 46.

GLENNER, G. G., TERRY, W., HARADA, M., ISENSKY, C. & PAGE, D. (1971) Amyloid Fibril Proteins: Proof of Homology with Immunoglobulin Light Chains by Sequence Analyses. *Science, N.Y.*, **172**, 1150.

JANIGAN, D. T. & DRUET, R. L. (1966) Experimental Amyloidosis. Role of Antigenicity and Rapid Induction. *Am. J. Path.*, **48**, 1013.

KUCZYNSKI, M. (1923) Weitere Beitrage zur Lehre von Amyloid. 3. Mitteilung (uber die Ruckbildung des Amyloids). *Klin. Wschr.*, **2**, 2193.

LEMONDE, P. (1959) Influence of Fighting on Leukemia in Mice. *Proc. Soc. exp. Biol. Med.*, **102**, 292.

LEMONDE, P. & MARGARIDA, C. (1962) Effect of BCG Infection on Leukemia and Polyoma in Mice and Hamster. *Proc. Soc. exp. Biol. Med.*, **111**, 739.

LUNDE, M. N. & GELDERMAN, A. H. (1971) Resistance of AKR Mice to Lymphoid Leukemia associated with a Chronic Protozoon Infection, *Besonitia jellisoni*. *J. natn. Cancer Inst.*, **47**, 485.

MISSMAHL, H. P. & HERTWIG, M. (1953) Polarisationsoptische Untersuchungen an der Amyloidsubstanz. *Virchows Arch. Path. Anat.*, **324**, 489.

PARR, I., WHEELER, E. & ALEXANDER, P. (1973) Similarities of the Anti-tumour Actions of Endotoxin, Lipid A and Double-stranded RNA. *Br. J. Cancer*, **27**, 370.

RANLØV, P. (1967) Humoral Immunity during the Induction of Experimental Amyloidosis. *Acta path. microbiol. scand.*, **69**, 375.

RANLØV, P. & JENSEN, E. (1966) Homograft Reaction in Amyloidotic Mice. *Acta path. microbiol. scand.*, **67**, 161.

RODNEY, G. E. & GOOD, R. A. (1969) Modification of the *in vitro* Response to Phytohemagglutinin of Mouse Spleen Cells by Amyloidogenic Agents. *Proc. Soc. exp. Biol. Med.*, **131**, 357.

ROWE, W. P. & PINCUS, T. (1972) Quantitative Studies of Naturally Occurring Murine Leukemia Virus Infection of AKR Mice. *J. exp. Med.*, **135**, 429.

SHEVACH, E., HERBERMAN, R., LIVERMAN, M., FRANK, M. M. & GREEN, J. (1972) Receptors for Immunoglobulin and Complement on Mouse Leukemias and Lymphomas. *J. Immun.*, **108**, 325.

STAATS, J. (1972) Standardized Nomenclature for Inbred Strains of Mice: Fifth Listing. *Cancer Res.*, **32**, 1609.

TEILUM, G. (1964) Pathogenesis of Amyloidosis. The Two-phase Cellular Theory of Local Secretion. *Acta path. microbiol. scand.*, **61**, 21.